# Multiperspectivity in organized sport in refugee sites: Sociological findings and pedagogical considerations

**DOI:** 10.3389/fspor.2022.1016010

**Published:** 2022-11-10

**Authors:** Enrico Michelini, Laura Schreiner

**Affiliations:** ^1^Laboratory of Sport Sociology and Economy, Economy and Sociology of Sport, Institute of Sport Sciences, University of Saarland, Saarbrücken, Germany; ^2^Department of Sports Science, Bielefeld University, Bielefeld, Germany

**Keywords:** refugee site, Sport for Development and Peace, multiperspectivity, systems theory, sport in refugee sites

## Abstract

Refugee sites are a means to manage large-scale refugee movements, a recurring event in today's world. Sport is supposed to have beneficial effects for the residents of such sites and is therefore an activity, which is standardly organized by the sites' management. Moreover, many NGOs and “Sport for Development and Peace” programmes endorse the use of sport in emergency situations—including in refugee sites—to achieve several biopsychosocial objectives. There is a growing body of scientific literature, however, that is calling into question the beneficial effects of sport in this setting as well as the rationale behind this idea. Against this background, we explore the question “How does multiperspectivity influence sport in refugee sites?” based on two case studies. We use the ethnographic materials we were able to collect for the case studies to conduct a (sociological) analysis of multiperspectivity in sport and to develop (pedagogical) proposals on how to incorporate multiperspectivity when devising sports activities for refugees. The fact that the perspectives and motivations beyond the actual sports activities in the refugee site setting might be extremely homogenous leads us to conclude that multiperspectivity is key for planning, organizing and monitoring sports activities, and that refugees' positions must also be acknowledged. We recommend programmes and practical ways of achieving these objectives with a focus on the role of trainers and coaches.

## Introduction

The term *refugee site* in the context of our study refers to a facility “built to provide immediate protection and assistance to people who have been forced to flee their homes due to war, persecution or violence” (1). It is used as an umbrella term for a wide range of settlements which includes but is not limited to camps. Such facilities are considered a *nomos* of our time (2) and play (ed) a crucial role in managing the “European refugee crisis” as well as the ongoing “Ukraine refugee crisis.” Despite their lifesaving function, refugee sites are a place of exception and generate limbo effects for residents due to the sites' mandatory spatiality and an often unknown duration of residency (3).

One of the most commonly accepted discourses, which is also heralded by the “Sport for Development and Peace” (SDP) movement, portrays sport as having huge potential to help, heal and empower refugees, including those who reside in refugee sites (4–7). Despite the existence of a large body of research on this topic, nearly all literature reviews (8–12) agree that the available research is subject to several limitations (5, 13–15): most of it hinges primarily on case studies or interventions that take this expectation as their starting point.

For this reason, numerous leading sociologists argue that the far-reaching potential attached to sport is widely overestimated (5, 13, 16–18). The ongoing efforts to produce evidence-based knowledge on SDP has accelerated the emergence of a community of scientists who assert that the belief in the utility of sport is faith-based (9). Despite the growing critical literature on and within SDP (4–6, 19), this remains in its core an applied the programme continues to be applied, which assumes the utility of sport and focuses on implementing and improving it.

Another important aspect is that the concept of SDP, even if well-intended, tends to embody a post-colonialist and hegemonic legacy, essentially reproducing established unbalanced power relations (20, 21). This imbalance of power tends to favor actors from the Global North (22, 23), which essentially means that many SDP projects are characterized by “donor-driven priorities” (24) and the target groups themselves have very little to no influence on project design (25, 26). Hence, “whilst a majority of these programmes are delivered in the Global South, many have been conceptualized in the Global North that is far removed from the realities as well as the inequalities being addressed” (27) (p. 589).

Therefore, recent international pedagogical literature on sport in refugee sites is mostly based on Freire's findings (17, 28–30) and other approaches to critical pedagogy (27, 31–33). While such pedagogic orientations are certainly ethically appropriate, they might also attach high and external expectations to sport (for example, of de-colonization and empowerment). That is, there might be again a disparity between the objectives and the actual potential of sport given the radical inequality refugees face and which refugee sites embody.

We argue that applying the concept of multiperspectivity may be a less “loaded” approach to developing a productive dialectic between all actors involved in sport and to emphasize refugees' needs. Building on our ethnographical observations, we suggest that recognizing the multivalent role of sport in refugee sites could represent the groundwork for fruitful pedagogical reflection on this particular setting. To this aim, we use “multiperspectivity” as our leading theoretical concept. Strongly inspired by social constructivism, the basic idea behind multiperspectivity applied to sport is that sport as *such* does not exist. Hence, sport is not a homogeneous phenomenon perceived and experienced in the same way by all actors. Instead, sport is a social construct that is largely co-created by the perspectives of its practitioners.

We explore the question “How does multiperspectivity influence sport in refugee sites?” After presenting our two case studies, we review the concept of multiperspectivity in depth and describe our methodological approach. Finally, we examine the multiperspectivity of sports activities in refugee sites and ways to approach sport in such settings at the programmatic and practical level. The advantages and pitfalls of developing meta-competences for incorporating multiple perspectives when devising sports activities in refugee site settings are discussed in the conclusion.

## Context—case studies

To limit the abstraction of the following discussion, this article uses two case studies on sport in refugee sites, which we review separately and independently of each other. Aside from the fact that both case studies are ethnographic analyses on sports activities in refugee sites, they cover different periods, locations, situations and (researchers') perspectives. Merging and comparing these two studies produces knowledge that is suitable for exploring our research question.

The first case study examined the organized sports activities provided through the Emergency Transit Mechanism (ETM) in Niger's capital city Niamey, a country in the Sahel region considered one of the least developed countries in the world (34). The ETM

“aims to provide life-saving protection, assistance and long-term solutions to extremely vulnerable refugees trapped in *detention* in Libya, through temporary evacuation to Niger. The aim is to deliver protection and identify durable solutions, including resettlement for these refugees, who are predominantly Eritrean and Somalian. Their profiles mainly include survivors of torture or other forms of violence in the country of origin and/or transit countries (e.g., Libya) and others with compelling protection needs. Many of them are unaccompanied children and women and girls at risk” (35).

While the ETM differs in nature from emergency and permanent camps, it nonetheless also generates limbo effects among the residents through its mandatory spatiality and a usually short albeit unknown and fluctuating duration of residency. In addition to providing refugees' livelihood^1^, the ETM also offers psychological support, language classes and sports activities, including football, swimming and Taekwondo for different groups and in diverse settings (within and outside of the sites). The sports activities were reviewed within the scope of a research project supported by UNHCR. The first author visited the ETM of Niamey for 4 weeks in 2019 and conducted participant observations of the sports activities offered at the sites. One of the major findings of this sociological study is that the sports activities provided at the ETM of Niamey embody different and sometimes conflicting goals and foci (36).

The second case study builds on data collected during a research visit to Amman, Jordan in 2020. The purpose of this visit was to review various SDP projects. Jordan has accommodated many refugees in recent decades. According to official figures, about 7% of Jordan's population are refugees (37). Unofficial estimations put this figure at nearly 15%. Moreover, there are over 2 million people who (or whose ancestors) are originally from the Palestinian territories; many are still not fully integrated in Jordanian society. The second author visited the Za'atari refugee camp, the largest in Jordan, during the visit to Amman. Za'atari was established in 2012 and is run by the Syrian Refugee Affairs Directorate and UNHCR (38). In 2020, about 80,000 people lived in the camp, more than half of whom were children. The conditions in Za'tari differ from those in the ETM of Niamey, specifically the temporal dimension. Many of the residents have been at the camp since its establishment and are expected to remain for some time to come. Many children were born in the camp and have already spent their entire lives there. Not least for these children, a variety of sports and play activities are offered at the camp, such as football and Taekwondo. The Za'atari camp visit took place on “International Women's Day,” hence not only was the camp itself visited, but the girls' sports groups also demonstrated their skills and a girls' football tournament took place.

Despite the fundamental differences between the refugee camp in Jordan and the ETM in Niger, the efforts and commitment witnessed at the respective sites were remarkable and the quality of the activities offered and the trainers' professionalism, engagement and passion were truly commendable. Given the extraordinary context within which the sports activities were being carried out, they felt quite “normal:” the activities were well-implemented and it was evident that the participants enjoyed them and were motivated. Despite these positive aspects, the potential of sport is clearly overestimated in both sites and several objectives are evidently being pursued through sport. In view of this, we first describe our socio-pedagogical approach before outlining our approach to data collection and analysis. The results section provides additional and more concrete information on the sports activities being offered at the two sites and considers their multiperspectivity based on the theoretical position discussed in the following section. While different sport disciplines are characterized by specificities and implications (39), the considerations of this study apply to sport in general.

## Theoretical framework

This study applies Luhmann's (40) systems theory to analyse the pedagogy-inspired concept of multiperspectivity of sport in refugee sites. This section explains our pedagogical and sociological approaches as well as their interconnections.

### Multiperspectivity

In the pedagogical approach, which was originally developed within the context of schools and first applied to sports science by Ehni (41) and Kurz (42), multiperspectivity basically implies that there are several perspectives for the same phenomenon (in our case, sport). Multiperspectivity is widely accepted in the German pedagogy of sport (43), but is described in different ways. Multiperspectivity can be understood with reference to different theoretical approaches, e.g., motivation theory (44) or action theory (45). Strongly inspired by social constructivism, the basic notion behind it is that diverse and variable subjective and collective stagings of sport exist. Sport is constructed and therefore perceived, evaluated and actively produced as a specific phenomenon that depends on different variables, including, but not limited to, setting, the subjects involved and their specific roles. Since not only one sport *per se* exists that is binding for all, it makes sense to design and research sport in a way that reflects the differences and similarities of people's perspectives of sport (44).

Building on this notion, the basic idea behind the concept of multiperspectivity is that different perspectives and meanings of sport exist, that sport is experienced differently by different persons (46) and that participants are motivated and motivate themselves based on multiple perspectives (47), i.e., “(a)s a characteristic, multiperspective means to look at something from different points of view, to deal with one thing under several perspectives” (47) (p. 149). Thus, different (pedagogical) intentions may be included in sports activities as well as in the multiple perspectives of its participants. At the same time, the concept opens up the possibility for both practical and reflective teaching content, whereby it is always possible to switch between perspectives (47). Accordingly, multiperspectivity is characterized by openness, connectability and expandability (46, 48).

However, it is “not a theoretically sharply defined, derived model” (47) (p. 150). Therefore, in the following, the perspectives that are of relevance in the context of refugee sites as well as the conditions that influence sport in refugee sites will be elaborated along systems theoretical considerations. Based on this, a proposal building on empirical data is presented which identifies and brings together perspectives that are relevant in refugee site settings. We aim to develop a model that can be used as a guiding framework for analyses of sport in refugee sites and that—in line with the concept of multiperspectivity—additional situationally relevant or meaningful perspectives can be added at any time (49).

### Systems theoretical considerations

By approaching multiperspectivity through systems theory, we intend to carry out a more refined and comprehensive analysis of sports activities offered at refugee sites. According to Luhmann, communication is the constitutive element of both society (40) (p. 63) and of social systems, which are self-referential, autopoietic and operationally closed aggregates of communication (40, 50). The different types of social systems are society with its functional subsystems, organization systems and interaction systems (51) (p. 24).

The process of functional differentiation represents a major (social) turning point in modern society. Examples of function systems are politics, mass media, education, health, economy, law, family and sport. These subsystems emerged as independent systems but are also closely coupled with the others by contributing to the (re-)production and integration of society (52). It should be noted that neither society nor function systems can be dominated by any one particular system, no matter how central its position is, as in the case of politics, health and the economy (53) (p. 131). Operationally closed systems cannot directly influence each other's operations within the meaning of a simplistic cause-effect relationship. Even in cases of close coupling, the autopoiesis of function systems implies a self-referential reproduction of their constitutive elements.

Sport is one of the many function systems that is differentiated in modern society (54–57). It is specialized in the communication of physical performances (58) (p. 380) through the code “victory/defeat” (57) (p. 185). From this viewpoint, sport communications interpret physical movements through a sports-related institutionalized vocabulary and focus (58) (p. 379) oriented toward competition. Sport descriptions transcend numerous features and details of sport events by reducing them into measurable and relevant information (58) (p. 379). This reduction not only ensures the possibility of understanding but also of comparing expected and completed sports performances, which in turn leads to the spread of communication (58) (p. 379). Sport is a multifaceted, complex and differentiated system that relies on privileged structural couplings with other systems, particularly with mass media, health, politics and the economy (59–61). Therefore, sport is not only limited to its specific systemic organizations, but is instead broadly organized in non-sport organizations such as schools, prisons and refugee sites as well.

Our research focusses on refugee sites. According to “camp studies” (3, 62, 63), refugee sites are spaces of exception defined by (blurred) boundaries and (supposed) temporariness. They can be used to destroy or to save lives and for many other goals in between these two extremes (repression, violence, segregation, hospitality, care, solidarity). Notably, social interactions in refugee sites are deeply influenced by the characteristic “liminality” (64) of the organization, creating a limbic setting. In the analysis, it is therefore not only important to identify the different functional and organizational systems that can influence the use of sport in refugee sites, but also to find out how this liminality comes into play, and further how it can be located in terms of systems theory. Agamben (2) describes refugees confined in reception sites as “homo sacer,” or as persons who are outside the law or beyond it. In this setting, they live a “bare life,” which “is included in the juridical order solely in the form of its exclusion” (2) (p. 12). For these reasons, the contradictory existence of camps and the vulnerability of their residents is considered a nomos of our time (2). The extension of the duration of residency in such camps is often frustrating and implies large availability of time, which is sometimes occupied with sport. Though not surprising, the availability of organized sport for refugees in such settings is not obvious (36). Indeed, refugee sites do not have an obligation to offer sports activities for residents and, in a context of endemic scarcity, any relevant resources could in fact be allocated elsewhere.

Interactions are a type of system that implies the co-presence of persons as a delimitation criterion. The ego/alter model of communication determines the subjectivity of face-to-face interactions (65) (p. 183). Refugees that reside in refugee sites are an extremely heterogeneous group (66). Nevertheless, refugee site operations entail “doing refugee” processes (67, 68)^2^. Some sociological studies suggest that the “dependency syndrome” affects the residents of such sites (69, 70), but this has been considered to be an illusion of the dominant class (43). Either way, it is important to acknowledge that despite their status and exceptional living conditions, refugees have agency and are not “apolitical, docile, dependent recipients who benefit enormously from humanitarian intervention” (71) (p. 29).

We argue that linking the concept of multiperspectivity with systems theory allows for considering different expectations, perspectives and intentions that are brought to sports in refugee sites. In this way, it is possible to combine different levels that become relevant in this form of use of sport in one model: The level of functional subsystems, each of which ascribes its own meaning to sport, as well as that of the organizational systems involved in the management in the context of the use of sport in refugee sites. In addition, at the level of interaction systems, the perspectives of various stakeholders, such as coaches or participants, i.e., their respective intentions or expectations, can be identified and included in the considerations. In other words, systems theory helps to identify a wide variety of relevant perspectives, while the concept of multiperspectivity helps to combine them into a model, which we believe is essential for the use of SDP in refugee sites.

## Methods

This study examines the multifaceted role of sport in refugee sites against the background of pedagogical and sociological reflections. It focusses on sport within the scope of UNHCR's ETM of Niamey based on data collected in 2019, as well as on data collected in 2020 on the Jordanian refugee camp Za'atari. In both cases, data were collected using ethnographic methods (72).

The first author participated in organized sports activities offered at the Niamey's ETM during a 4-week research visit. The research activities included observations, interviews and complementary forms of data collection. Approximately 60 h of participant observations were spent actively taking part in sports activities with refugees, some of these hours as one of the coaches in a football-based project (20 h) and the rest (40 h) as an active participant in other sports activities offered at the sites (swimming and Taekwondo). An equal amount of time was spent at the ETM before and after participation in the given sports activities. Exchanges before, during and after the sports activities, including conversations, answers to individual questions and informal chats with refugees and with people working at the sites also took place. Interviews were another relevant source of information. Ten narrative interviews focussed on the topic of sport in Niamey's refugee sites and were conducted with staff working at the sites. The interviewees were three interpreters who had a refugee background, four managers with European roots employed with UNHCR and three trainers from Niger. Each of the interviews lasted around 1 h and were carried out formally on appointment after having informed the participants about the study's objectives and after obtaining their consent. Finally, complementary information was collected through document analysis. Documents in this regard are understood as any piece of information (image, text, audio or video) that are available independently from the study's research activities. The materials on sports activities issued by the organizations, journalists or individuals involved in making the activities possible were considered complementary sources.

The second author collected data within the framework of a (participatory) ethnographic observation. The author visited and observed certain sections of the camp, day-to-day life as well as the sports activities that took place on the day of the visit to the Za'atari camp, namely on “International Women's Day.” The activities included a performance of the girls' Taekwondo group as well as a football tournament of Za'tari's girls' football groups. The camp's male residents were not allowed to participate in or watch the games that day. In addition, exchanges with the participants in sports activities as well as with employees of various aid organizations and a state organization in charge of implementing projects in Za'atari took place before, after and during the visit. Both observations and the relevant content of the conversations were recorded in writing from memory.

In both cases, the data collected through these methods consisted of field notes and diaries, transcriptions and document analyses. After the visits, the notes and diaries were integrated and adapted, but not reworked or interpreted. These data were used as groundwork for reflection and comparison of the authors' experiences, particularly their common observations of sport's multiperspectivity. The material was analyzed using content analysis techniques (73) and interpreted based on the sociological and pedagogical perspective discussed above.

## Results

The following sections focus on sport's multiperspectivity by examining the different societal levels mentioned above and elaborating Luhmann's (51, 74) typology of systems.

Different systemic, organizational and interactional logics play a relevant role and influence the features of organized sports activities in refugee sites. Moreover, the (situational) conditions of the respective sites must be considered as well, for example, with regard to the state of emergency or the available resources. Our study considers the participants and their individual perspectives to be central to the given sports activities. After all, they are our main target and are participants in the sports activities.

### Society

Societies are encompassing systems that include all possible forms of communication. Modernity is characterized by the emergence of one encompassing world society and by the differentiation of function systems within society (65) (p. 183). However, the refugee sites are marked by liminality (64) and exceptionality (2). This implies that the lifeworld of the residents of such sites is characterized by radical exclusion, boredom and limited resources and access to the outside world.

Despite this isolation, the influence of systemic logics is visible, also with regard to the physical activities being offered. While the distinction is not clear-cut, the relevance of different systemic logics and multiple perspectives was evident and will be explained below.

First, sport is a major pillar of recreational activities. Besides being both a way to kill time and a form of play, elite sport is also a source of inspiration for young refugees. The dream of pursuing a professional football career in Europe is a recurrent *topos* of interviews with migrants at refugee sites, for example (75). At the sites themselves, sport is mostly carried out in its essence of playful physical activity oriented toward performance. In this form, the activities are very similar to sport club activities.

Second, the educational logic is evident with reference to the aim of not only teaching sport but also of different soft and social skills through sport. Sport is implemented as a physical education class to achieve different goals, for example, gender equality and discipline. Taekwondo is used in the Za'atari camp to empower girls, to teach them self-confidence but also to teach them skills self-defense and self-assertion, thus improving their overall safety. In the ETM of Niamey, the same sport is primarily practiced by male youth, with the aim of teaching them (self-) respect and discipline rather than simply being a form of martial arts.

Third, sport is sometimes used as a medicine. Sport in this sense is a way of providing medical care to refugees, which is in line with the core humanitarian goal of the refugee site setting. In this regard, sports activities themselves are perceived as an activity to be promoted because it has the potential of strengthening the bio-psycho-social health of its participants. For example, football programmes aim to improve participants' health while swimming is assumed to have therapeutic effects.

The use of sport to pursue other goals might be the key to understanding the relevance of sport in refugee sites and the widespread diffusion of SDP programmes. At the meta-level, sport is also a means of power used to control, distract and discipline the residents of refugee sites and is therefore a way to enforce the power of the organization and its staff over refugees. In this regard, sport fosters unbalanced power relations but also contributes to the self-reproduction of the organization itself, as argued in the following section.

### Organization

Organizations emerge as communication systems with the capacity to stabilize types of action and behavior through decisions taken on the basis of specific premises and organizational cultures (65) (p. 183). Refugee sites are humanitarian organizations specialized in the management of forced migration (66). Decisions on refugee sites are guided by a mix of systemic logics (76, 77), which include law (legal/illegal), political (inferior/superior power) and moral logics (right/wrong). However, its decisions are not only guided by structural couplings between these sometimes contradictory logics but also by states of emergency, scarcity of resources and the direct involvement of many political and humanitarian organizations. At the ETM of Niamey, UNHCR is involved as the leading operator along with other UN agencies and NGOs in the sites' management. Additionally, sports organizations, for example, the foundations of two professional football teams, contribute financially as well as with material and knowledge to the organization of activities. At the Za'atari camp, various organizations provide materials or train (future) coaches through “train-the-trainer” programmes, including, for example, NGOs and state organizations, such as the *Deutsche Gesellschaft für internationale Zusammenarbeit* (GIZ). In terms of the organization of sport, this implies that the power is divided between different organizations that have diverse perspectives and diverging sport know-how.

When considering organizations, systems theoretical literature traditionally focusses on the following decision premises (76, 78, 79): decision programmes define the organization's goals and how to achieve them. Communication channels are horizontal and vertical divisions of tasks, hierarchies and assignments of responsibilities concerning work processes. Human resources refers to how individuals are assigned to different areas of action and responsibility by identifying and matching their qualifications. While an analysis of communications is less feasible on the basis of our available data, and as the topic of human resources will be assessed in the next session, this one focusses on programmes.

At the programmatic level, UNHCR borrows positions and goals from SDP to carry out sports activities at the ETM^3^. From the perspective of SDP, sport broadly consists of “*all forms of physical activity that contribute to physical fitness, mental wellbeing and social interaction. These include play; recreation play; recreation; organized, casual or competitive sport; and indigenous sports or games*” (80) (p. 2). Sport is expected to produce relevant bio-psycho-social benefits (81) for refugees residing in refugee sites because

“*(m) any of the core values inherent in sport are compatible with the principles necessary for development and peace, such as fair play, cooperation, sharing and respect. The life skills learned through sport help empower individuals and enhance psychosocial wellbeing, such as increased resiliency, self-esteem and connections with others. These features of sport are beneficial to people of all ages, but they are especially vital to the healthy development of young people”* (80) (p. 2).

Specifically for refugees, “(t)he psychosocial benefits from the practice of sport help to address the trauma of flight and the distress resulting from displacement. Sports programmes serve as a positive and productive activity for refugees and internally displaced persons, easing many of the problems they face, including violence, limited access to education and broken family structures.” (80) (pp. 15-16). The UN acknowledges that sport, “like many aspects of society, simultaneously encompasses some of the worst human traits, including violence, corruption, discrimination, hooliganism, excessive nationalism, cheating and drug abuse” (80) (p. 2). Despite this relativisation, the expectations of sport at the programmatic level are very high, particularly concerning the goal of empowerment because the residents of refugee sites are by definition a radically disempowered population group.

Moreover, the application of the guidelines mentioned above need to necessarily be adapted to the specific situation of the given refugee site. The ETM in Niger is embedded in a socio-political context, which plays a relevant role though an in-depth analysis exceeds the aims of this study. More importantly, the poverty in the country also implies an endemic scarcity of resources that needs to be compensated by UNHCR, the main actor in this setting. The sub-Saharan climate poses an additional challenge for sport. The Za'atari camp, on the other hand, is characterized by refugees' long duration of stay. The camp was initially not planned as a refugee camp, but rather developed from a tent camp after the first refugees arrived there. In the meantime, however, most of the residents have lived there for years. For the children, this means that a large part of their education takes place at the camp, and at the same time, a balance must be created. Sport is meant to contribute in particular to community empowerment and the education of livelihoods (82). At the same time, it must be remembered that only 18% of refugees in Jordan actually live in refugee camps (37). The challenge is therefore to provide for all refugees equally.

### Interaction

Interaction is a type of system that implies the co-presence of persons as a delimitation criterion. The ego/alter model of communication determines the subjectivity of face-to-face interactions (65) (p. 183). The participants themselves, the trainers and managers/organizers come together at the interaction level.

At the ETM, the various actors directly involved in organizing and implementing sports activities have different perspectives on sport. Amongst the most influential actors involved in the organization and/ or implementation of the activities are the mangers, trainers and participants. Their interactions are mediated by the environmental factors (discussed above).

The managers' perspective is particularly relevant despite the fact that they are not direct participants in the activity. Their decisions are crucial for the legitimacy, realization and maintenance of the sports activities. At the ETM, the managers/organizers are international staff of UNHRC or of other NGOs. Aside from providing training programmes for trainers and the necessary funds, they also bring their own organizational perspective, in that sport is intended to achieve specific (organizational) goals. Amongst the goals borrowed from SDP for carrying out sports activities, health goals are the main reason mentioned by ETM managers. Other reasons include education, discipline and leisure.

The trainers clearly influence the sports activities that are provided at the ETM. All of them were locals^4^ who had been professional athletes in the past and had sport club socialization and training. Despite referring to the reasons also cited by management (it is impossible to establish whether genuinely or strategically), they implemented the activities using many classic elements of competitive sport.

At the Za'atari camp, the trainers were often refugees and former participants of sports activities offered there as well as of so called “train-the-trainer” programmes, which train future trainers in how to work with children. The main focus of the programmes observed in Za'atari was the empowerment of girls. Accordingly, the programmes were a mixture of sports-oriented content and content aimed at strengthening the girls' soft skills and personality. It was striking that the sports facility was completely shielded from the outside to create a protected space for the girls and women. The sports classes on the day of the visit took place within that space, although there were both male and female visitors that day.

At the ETM, the sites' residents take part in one or more organized sports activities. Most of them “are predominantly Eritrean and Somalian. Their profiles mainly include survivors of torture or other forms of violence in the country of origin and/or transit countries (e.g., Libya) and others with compelling protection needs. Many of them are unaccompanied children and women and girls at risk” (35). As refugees are a highly heterogeneous group—even within the selected target group of the ETM—it is impossible to generalize the goals of their participation, which range from social to sportive and everything in-between. However, killing time was a recurrent motivation for participating in sports activities. At the Za'atari camp, the participants—they were girls, at least within the scope of the SDP projects observed—bring in their own perspectives (e.g., their own experiences, wishes and needs); often, their direct caregivers (such as their parents) also have considerable influence on their choices. This might then determine which type of sport they prefer to engage in or which goals they seek to achieve by engaging in sport.

Working in the field of sport with refugees can be very inspiring, fulfilling and fun for the trainers, but may also cause frustration (84–86). Likewise, sport can benefit refugees but may also cause stress, discomfort and re-traumatisation (87, 88). The setting implies that progress may be swift but ambitious goals may be unattainable. Alone engaging in a well-frequented, fun and conflict-free activity is a basic yet compelling goal. Therefore, sport is only seemingly a sanctuary where opportunities for recreation are offered to refugees. In refugee sites, sport becomes something extraordinary and multivalent, but also elusive and ambiguous. Perhaps sport nourishes a physicality that characterizes refugees' bare life (2). Thereby, the body, satisfying its needs, and the preservation of its deriving capital (89) are some of the remaining fixed points and achievable goals for refugees at certain points of their lives.

## Conclusion and discussion

A sociological and pedagogical analysis of the multiperspectivity of sport in refugee sites was carried out based on two case studies. To this aim, the main concepts of sport and their application to the specific contexts of the ETM of Niamey and the refugee camp Za'atari were explored. Organized sport plays an important role at both sites and is guided by multiple meanings that rely on the logics of sport, health and education; are influenced by the decision premises of the leading organizations; and are further mediated by the sometimes conflictual interactions of the actors involved.

**Figure 1:**
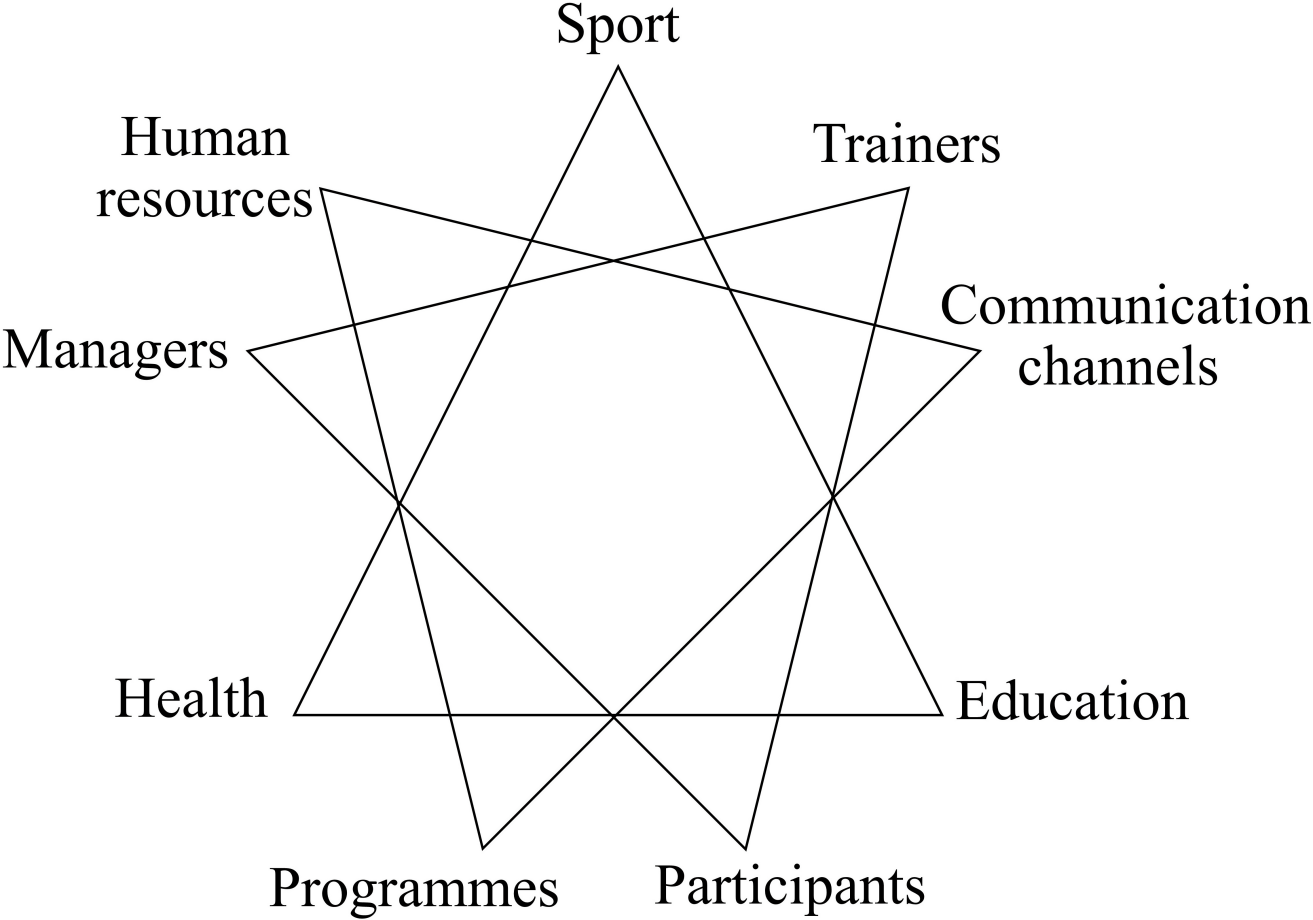
Main perspectives on sport.

Our analyses confirm that sport pursues different goals and has different foci (36), which rest on macro-, meso- and micro factors. An analytical description of the perspectives on sport is compelling because they overlap and are consciously (or not) staged or concealed. Our study presents an analytical description based on types of systems, namely society, organization and interaction (see Figure 1). These perspectives on sport entail strengths and weaknesses and may be more or less suitable for implementing sports activities. Moreover, this mix of perspectives implies that the meaning of sport in refugee sites is multifaceted yet also elusive and loaded with expectations, which are sometimes utopic. This inextricable mix of meanings may also create discrepancies between the goals, planning and implementation of sports activities and their perception at the individual level.

This conclusion reflects a general consideration of multiperspectivity, which may help organizations, trainers and participants better understand and relate their own and others' perceptions, attitudes and goals in sport. This, in turn, may foster a convergence of the multitude of perspectives, thereby mitigating contradictions and preventing conflict.

When considering the threats and opportunities that emerge from the multiperspectivity of sport in refugee site settings, recommendations can be formulated through pedagogical lenses. While such recommendations can be address different levels and actors, our study specifically targets trainers, because they can more easily and directly adapt sports activities. Multiperspectivity is a key meta-competence to better understand, reflect on and purposefully manage sport in refugee camps. Pushing through one's own ideas on and perceptions of sport, which is frequently observed in sport pedagogy (90), bears risks. Moreover, the inclusion of foreign goals entails the risk of only superficially or cursorily working with them. Finally, the capacity to empathize with others whose cultural and legal status differs profoundly from one's own cannot be taken for granted. Desensitization processes are well-documented in studies on humanitarian workers (83). Developing such meta-competence is therefore crucial to help trainers deliver sports activities that are adapted to the specific context, to take all perspectives and expectations into consideration and to ensure that the goals are aligned with the perspectives of the refugees. After all, they are the target of the activities and are at the same time in the weakest position.

Because sport in refugee sites is multifaceted as well as elusive and loaded with expectations, trainers should be well-aware of the goals they are pursuing and ask themselves [modified from Eikenberry (91)]: Who is my target group as a trainer? Which goals are appropriate for my target group? Why is achieving this goal important? What outcome do I expect to achieve? How will the refugees benefit from reaching this goal? What is the first step? What step will take me the furthest, fastest? Who can help me achieve it? Who will support me? Who will not support me?

**Figure 2:**
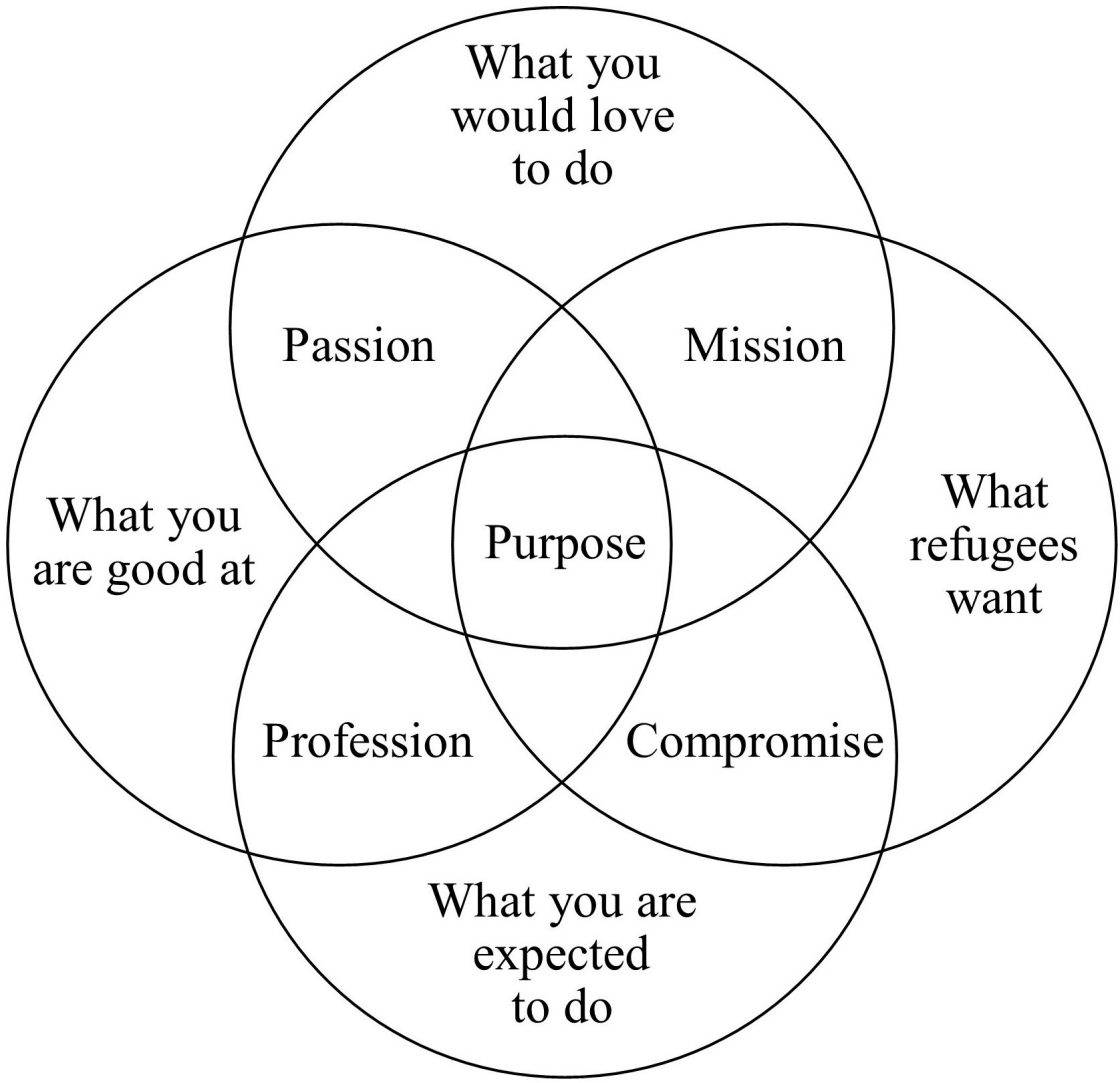
Venn diagram for sport in refugee sites.

Identifying and reflecting on goals connected to multiple perspectives should be encouraged among trainers by implementing specific activities. Discussions on the topic of multiperspectivity with staff and with other trainers as well as reviewing other sports activities are simple and feasible ways of encouraging a reflective approach to this issue. The intervention of an external consultant and the use of specific tools such as the Venn diagram developed by the authors (Figure 2) could help stimulate deep and fruitful reflection. This situation is however complicated by further variables, which need to be considered when reflecting on perspectives for setting goals. The context and the various sport disciplines have diverse implications, which need to be constantly reflected on. Importantly, trainers need to be motivated, dedicated and aware of the implications of conducting sports activities for refugees in refugee sites.

Sensibly dealing with multiperspectivity might also be an option for dealing with empowerment, a meta-goal that is central in the SPD's discussion. Empowering refugees by encouraging self-organization in sport is an often quoted but in practice often disregarded, ambitious and meaningful goal. In line with Giulianotti and Armstrong (92) (p. 16), we exclude the possibility that sport *per se* can heal, educate or empower refugees. The idea of empowering residents of a refugee site in a highly disempowering setting is in itself contradictory or at the very least challenging. Therefore, sport needs to be carefully planned, implemented and controlled to foster empowerment. In the SDP, sport has been used among other aims to provide “an enjoyable recreational space in which groups may build a stronger public presence and social role, enjoy healthy and playful exercise, and improve substantially their self-esteem and confidence” (92) (p. 20). Empowerment is “the capacity of individuals, groups and/or communities to take control of their circumstances, exercise power and achieve their own goals, and the process by which, individually and collectively, they are able to help themselves and others to maximize the quality of their lives” (93) (p. 6). One concrete way of fostering empowerment is supporting the autonomous and sustainable implementation of sports activities by refugees themselves. While research (94) indicates that this is by no means an easy feat in the context of refugee sites, refugees could develop and manage their own sports activities if they received the necessary freedom, help and support. This, in turn, can create processes of individual and collective responsibilisation, increase the number of sports activities being offered as well as the number of participants. It may even constitute vocational training for some of them. While this is indubitably a long and difficult process, taking different perspectives into account and giving them room, for example, by letting refugees choose a sports activity or the focus of the sessions, could be first practical steps. This should be followed by a progressive transfer of responsibilities—and thus power—with the ultimate aim of refugees self-managing sport. The authors' experiences suggest that this goal is everything but impossible, and the biggest hurdles are embedded in power-related organizational processes rather than in the refugees' competence and engagement. Finally, the Anishinaabekweg commitment to decolonisation through physical activity is an example of how personal decolonisation through physical activity can be achieved in a way that is rooted in refugee's self-identified needs, knowledge and cultural practices (95).

To conclude, we do not challenge the fact that a critical implementation of SDP must be “historically and geographically informed both with regard to development inequality and to the social and political dimensions of sport” (5). However, refugee sites are embedded in a local socio-political and cultural situation, and yet is also a place of exception, with strong intrinsic dynamics. Therefore, we argue that the participants' individual needs should play an (even more) central role in pedagogical practice, which can be supported by multiperspectivity.

By re-analyzing and comparing the results of our two ethnographic studies through a pedagogical lens, this article moved to an intermediate step between a descriptive and an intervention-oriented research study. The recommendations are still too abstract to be concretely implemented. To be able to arrive at more concrete conclusions, a follow-up longitudinal study in different refugee sites should be carried out, which also entails the possibility of influencing the sports activities. Such a project would also resolve the limitations of the discussed ethnographic studies (96), which were limited in time and compared a posteriori. Such a follow-up project should also aim to establish a deeper contact with the refugees, which in the present study were a less accessible data source.

## Data availability statement

The raw data supporting the conclusions of this article will be made available by the authors, without undue reservation.

## Ethics statement

The studies involving human participants were reviewed and approved by TU Dortmund's Ethics Committee. Written informed consent for participation was not required for this study in accordance with the national legislation and the institutional requirements.

## Author contributions

EM was responsible for the conception and writing for the article. LS contributed by sharing data on the second case study, by contributing in the data analysis, interpretation. All authors contributed to the article and approved the submitted version.

## Funding

We acknowledge support by the Deutsche Forschungsgemeinschaft (DFG, German Research Foundation) and Saarland University within the Open Access Publication Funding programme.

## Conflict of interest

The authors declare that the research was conducted in the absence of any commercial or financial relationships that could be construed as a potential conflict of interest.

## Publisher's note

All claims expressed in this article are solely those of the authors and do not necessarily represent those of their affiliated organizations, or those of the publisher, the editors and the reviewers. Any product that may be evaluated in this article, or claim that may be made by its manufacturer, is not guaranteed or endorsed by the publisher.

## Footnotes

^1^All sites provide inhabitants with water, food, medicine, shelter and clothing.

^2^“Doing gender” is a term coined by West and Zimmerman (68) in their homonymous article. It refers to the idea that gender is not an innate quality of individuals and is instead “a routine, methodical, and recurring accomplishment” (68) (p. 126). Similarly, “refugee” is a label that creates expectations and obligations, constituting a rigid accountability structure. On this basis, refugees are judged and judge themselves in terms of “good/bad,” “successful/unsuccessful,” “included/excluded”.

^3^Further related concepts can be consulted in the *Sport for Development and Peace: Toward Achieving the Millennium Development Goals* (80).

^4^While reasons against recruiting trainers from among the camp's inhabitants exist, refugees would clearly be capable of managing their own sports activities (and much more) among themselves (83) (p. 57).

## References

[1] UNHCR. What is a Refugee Camp? (2020). Available online at: https://www.unrefugees.org/refugee-facts/camps/ (accessed July 18, 2022).

[2] Agamben G. Homo Sacer: Sovereign Power and Bare Life. Stanford: Stanford University Press (1998).

[3] Turner S. What is a refugee camp? Explorations of the limits and effects of the camp. J Refugee Studies. (2016) 29:139–48. 10.1093/jrs/fev024

[4] Collison H Darnell SC Giulianotti R Howe PD. Routledge Handbook of Sport for Development and Peace. New York, NY: Routledge (2019).

[5] Darnell SC. Sport for Development and Peace: A Critical Sociology. New York, NY: Bloomsbury (2012).

[6] Schulenkorf N Adair D. Global Sport-for-Development: Critical Perspectives. London: Palgrave Macmillan (2013).

[7] Young K. Sociology of Sport: A Global Subdiscipline in Review. Bingley: Emerald Group (2017).

[8] Cronin O. Comic Relief Review: Mapping the Research on the Impact of Sport and Development Interventions (Vol. Manchester). Manchester: Orla Cronin Research (2011).

[9] Langer L. Sport for development – a systematic map of evidence from Africa. South Afric Rev Soc. (2015) 46:66–86. 10.1080/21528586.2014.989665

[10] SDP International Working Group. Literature Reviews on Sport for Development and Peace. Toronto: Univeristy of Toronto (2007).

[11] Svensson PG Woods H. A systematic overview of sport for development and peace organisations. J Sport Dev. (2017) 5:36–48.

[12] van Eekeren F ter Horst K Fictorie D. Sport for Development: the Potential Value and Next Steps: Review of Policy, Programs and Academic Research 1998-2013. Utrecht: International Sports Alliance (2013).

[13] Darnell SC. Power, politics and“ sport for development and peace”: investigating the utility of sport for international development. Sociol Sport J. (2010) 27:54–75. 10.1123/ssj.27.1.54

[14] Giulianotti R. The sport, development and peace sector: a model of four social policy domains. J Soc Policy. (2011) 40:757–76. 10.1017/S0047279410000930

[15] Kidd B. A new social movement: sport for development and peace. In: Jackson SJ, HaighS, editors. Sport and Foreign Policy in a Globalizing World. London: Routledge (2013). p. 36–46.

[16] Mwaanga O. Reconceptualizing sport for development and peace (SDP): an ideological critique of Nelson ‘Madiba' Mandela's engagement with sport. Sport Soc. (2020) 23:847–63. 10.1080/17430437.2019.1584184

[17] Spaaij R Jeanes R. Education for social change? A freirean critique of sport for development and peace. Physic Educ Sport Pedagogy. (2013) 18:442–57. 10.1080/17408989.2012.690378

[18] Wright RW. Understanding the Role of Sport for Development in Community Capacity Building in a Refugee Camp in Tanzania. Saskatoon: University of Saskatchewan (2009).

[19] Young K Okada C. Sport, Social Development and Peace: Emerald Group Publishing (2014).

[20] Darnell SC Hayhurst LMC. Sport for decolonization: exploring a new praxis of sport for development. Prog Dev Studies. (2011) 11:183–96. 10.1177/146499341001100301

[21] Hartmann D Kwauk C. Sport and development an overview, critique, and reconstruction. J Sport Soc Issues. (2011) 35:284–305. 10.1177/0193723511416986

[22] Lindsey I Banda D. Sport and the fight against HIV/AIDS in Zambia: a 'partnership approach'? Int Rev Sociol Sport. (2011) 46:90–107. 10.1177/1012690210376020

[23] Welty Peachey J Cohen A Shin NR Fusaro B. Challenges and strategies of building and sustaining inter-organizational partnerships in sport for development and peace. Sport Manag Rev. (2017) 21:160–75. 10.1016/j.smr.2017.06.002

[24] Nicholls S Giles AR Sethna C. Perpetuating the 'lack of evidence' discourse in sport for development: privileged voices, unheard stories and subjugated knowledge. Int Rev Sociol Sport. (2011) 46:249–64. 10.1177/1012690210378273

[25] Giulianotti R Coalter F Collison H Darnell SC. Rethinking sportland: a new research agenda for the sport for development and peace sector. J Sport Soc Issues. (2019) 43:411–37. 10.1177/0193723519867590

[26] Hayhurst LM. The power to shape policy: charting sport for development and peace policy discourses. Int J Sport Policy Politics. (2009) 1:203–27. 10.1080/19406940902950739

[27] Mwaanga O Prince S. Negotiating a liberative pedagogy in sport development and peace: understanding consciousness raising through the go sisters programme in Zambia. Sport Educ Soc. (2016) 21:588–604. 10.1080/13573322.2015.1101374

[28] Nols Z Haudenhuyse R Spaaij R Theeboom M. Social change through an urban sport for development initiative? Investigating critical pedagogy through the voices of young people. Sport Educ Soc. (2019) 24:727–41. 10.1080/13573322.2018.1459536

[29] Oxford S Spaaij R. Critical pedagogy and power relations in sport for development and peace: lessons from Colombia. Third World Thematics. (2017) 2:102–16. 10.1080/23802014.2017.1297687

[30] Spaaij R Oxford S Jeanes R. Transforming communities through sport? Critical pedagogy and sport for development. Sport Educ Soc. (2016) 21:570–87. 10.1080/13573322.2015.1082127

[31] Luguetti C Singehebhuye L Spaaij R. Towards a culturally relevant sport pedagogy: lessons learned from African Australian refugee-background coaches in grassroots football. Sport Educ Soc. (2020) 1–13. 10.1080/13573322.2020.1865905. [Epub ahead of print].

[32] Luguetti C Singehebhuye L Spaaij R. ‘Stop mocking, start respecting': an activist approach meets African Australian refugee-background young women in grassroots football. Qualit Res Sport Exerc Health. (2021) 1–18. 10.1080/2159676X.2021.1879920. [Epub ahead of print].

[33] Mwaanga O Banda D. A postcolonial approach to understanding sport-based empowerment of people living with HIV/AIDS (PLWHA) in Zambia: the case of the cultural philosophy of Ubuntu. J Disabil Relig. (2014) 18:173–91. 10.1080/23312521.2014.898398

[34] UN. Human Development Report. New York, NY: UN (2022).

[35] UNHCR. Country Operation Update. (2019). Available online at: http://reporting.unhcr.org/sites/default/files/UNHCR%20Niger%20Operational%20Update%20-%20May%202019.pdf (accessed July 18, 2022).

[36] Michelini E. Organised sport in refugee sites: an ethnographic research in niamey. Eur J Sport Soc. (2022) 19:1–17. 10.1080/16138171.2021.1878433

[37] UNHCR. Jordan: Zaatari Refugee Camp. (2022). Available online at: https://www.unhcr.org/jo/wp-content/uploads/sites/60/2022/02/1-Zaatari-Fact-Sheet-January-2022-final.pdf (accessed July 18, 2022).

[38] UNHCR. Hilfe für Flüchtlinge in Jordanien. Folgen des Syrien-Konfliktes. (2022). Available online at: https://www.uno-fluechtlingshilfe.de/hilfe-weltweit/jordanien (accessed July 18, 2022).

[39] Korsik A Ivarsson V Nakitanda OA Perez Rosas L. Implementing Sports in Refugee Camps. Lausanne: AISTS (2013).

[40] Luhmann N. The self-description of society: crisis fashion and sociological theory. Int J Comp Sociol. (1984) 25:59–72. 10.1163/156854284X00052

[41] Ehni HW. Sport und Schulsport: Didaktische Analysen und Beispiele aus der Schulischen Praxis. Schorndorf: Hofmann (1977).

[42] Kurz D. Elemente des Schulsports. Schorndorf: Hofmann (1977).

[43] Reid C Al Khalil A. Refugee cosmopolitans: disrupting narratives of dependency. Social Alternatives. (2013) 32:14–9.

[44] Ruin S. Mehrperspektivität als sportpädagogischer Gemeinplatz? Eine konzeptionelle standortbestimmung. German J Exerc Sport Res. (2019) 49:127–39. 10.1007/s12662-018-0564-6

[45] Ehni HW. Sportunterricht in den perspektiven des handelns und erlebens. In: Neumann P, Balz E, editors. Mehrperspektivischer Sportunterricht. Orientierungen und Beispiele. Schorndorf: Hofmann (2004). p. 34–56.

[46] Neumann P. Einführung: mehrperspektivischer sportunterricht. In: Neumann P, Balz E, editors. Mehrperspektivischer Sportunterricht. Orientierungen und Beispiele. Schorndorf: Hofmann (2010). p. 7–18.

[47] Balz E Neumann P. Mehrperspektivischer sportunterricht. In: Aschebrock H, Stibbe G, editors. Didaktische Konzepte für den Schulsport. Aachen: Meyer and Meyer (2013). p. 148–177.

[48] Balz E. Perspektiven als bildungskategorien. In: Krüger M, Neuber N, editors. Bildung im Sport. Beiträge zu einer zeitgemäßen Bildungsdebatte. Wiesbaden: VS (2011). p. 179–186.

[49] Balz E. Perspektivisch unterrichten: didaktisch-methodische anregungen. In: Neumann P, Balz E, editors. Mehrperspektivischer Sportunterricht. Didaktische Anregungen und Praktische Beispiele. Schorndorf: Hofmann (2011). p. 53–66.

[50] Maturana HR Varela FJ. The Tree of Knowledge: The Biological Roots of Human Understanding. Boston: Shambhala (1987).

[51] Luhmann N. Schriften zur Organisation 2. Wiesbaden: Springer Fachmedien (2019).

[52] Luhmann N. The autopoiesis of social systems. J Soc. (2008) 6:84–95.

[53] Luhmann N. The world society as a social system. Int J General Systems. (1982) 8:131–8. 10.1080/03081078208547442

[54] Bette K-H. Körperspuren: zur Semantik und Paradoxie Moderner Körperlichkeit. 2 Ed. Berlin: de Gruyter (1989).

[55] Cachay K. Sport und Gesellschaft: Zur Ausdifferenzierung einer Funktion und ihrer Folgen. Schorndorf: Hofmann (1988).

[56] Cachay K Thiel A. Soziologie des Sports: Zur Ausdifferenzierung und Entwicklungsdynamik des Sports der modernen Gesellschaft. Weinheim: Juventa-Verlag (2000).

[57] Schimank U. Die entwicklung des sports zum gesellschaftlichen teilsystem. In: Mayntz R, editors. Differenzierung und Verselbständigung: Zur Entwicklung gesellschaftlicher Teilsysteme. Frankfurt: Campus (1988). p. 181–232.

[58] Stichweh R. Sport-ausdifferenzierung, funktion, code. Sportwissenschaft. (1990) 20:373–89.

[59] Bette K-H Schimank U. Doping im Hochleistungssport : Anpassung durch Abweichung 2nd Ed. Frankfurt: Suhrkamp (2006).

[60] Schimank U. Die gesellschaftliche entbehrlichkeit des spitzensports und das dopingproblem. In: Digel H, Editor. Spitzensport – Chancen und Probleme. Schorndorf: Hofmann (2001). p. 12–25.

[61] Schimank U. Sport im prozess gesellschaftlicher differenzierung. In: Weis K, Abraham A, editors. Handbuch Sportsoziologiez. Schorndorf: Hofmann (2008). p. 68–74.

[62] Martin D Minca C Katz I. Rethinking the camp: on spatial technologies of power and resistance. Prog Hum Geogr. (2020) 44:743–68. 10.1177/0309132519856702

[63] Ramadan A. Spatialising the refugee camp. Transactions. (2013) 38:65–77. 10.1111/j.1475-5661.2012.00509.x

[64] Ager A Strang A. Understanding integration: a conceptual framework. J Refug Stud. (2008) 21:166–91. 10.1093/jrs/fen016

[65] Nassehi A. Organizations as decision machines: niklas luhmann's theory of organized social systems. Soc Rev Monog. (2005) 53:178–91. 10.1111/j.1467-954X.2005.00549.x

[66] Malkki LH. Refugees and exile: from 'refugee studies' to the national order of things. Annu Rev Anthropol. (1995) 24:495–523. 10.1146/annurev.an.24.100195.002431

[67] Schütte D. Die eigenen verstrickungen reflektieren. In: Kaufmann ME, Otto L, Nimführ S, Schütte D, editors. Forschen und Arbeiten im Kontext von Flucht: Reflexionslücken, Repräsentations- und Ethikfragen. Wiesbaden: Springer (2019). p. 21–43.

[68] West C Zimmerman DH. Doing gender. Gender Soc. (1987) 1:125–51. 10.1177/0891243287001002002

[69] Abdi AM. In limbo: dependency, insecurity, and identity amongst somali refugees in dadaab camps. Refuge Cana J Refugees. (2005) 22:6–14. 10.25071/1920-7336.21328

[70] Boesen IW. From autonomy to dependency: aspects of the 'dependency syndrome' among afghan refugees. Migrat Today. (1985) 13:17–21.

[71] Besteman C. Making Refuge. Somali Bantu Refugees and Lewiston, Maine. Durham: Duke University Press (2016). 10.1215/9780822374725

[72] Jackson MA. Process and Emergence: A Topographic Ethnography of the Embodiment of Place and Adventure Tourism in Khumbu, Nepal. Ann Arbor, MI: Prescott College (2017).

[73] Krippendorff K. Content Analysis: An Introduction to its Methodology 3 Ed. London: Sage (2013).

[74] Luhmann N. Soziale Systeme: Grundriß einer allgemeinen Theorie. Frankfurt am Main: Suhrkamp (1987).

[75] Elia A Fedele V. (Unaccompanied) young migrants and football: mediating a'safe space'for enhancing subjectivities systems. Ital Soc Rev. (2021) 11:691–714.

[76] Luhmann N. Organisation und Entscheidung 2 Ed. Wiesbaden: VS (2006).

[77] Roth S Schutz A. Ten systems: toward a canon of function systems. Cybernetics Human Knowing. (2015) 22:11–31. 10.2139/ssrn.2508950

[78] Thiel A Mayer J. Characteristics of voluntary sports clubs management: a sociological perspective. Eur Sport Manag Quart. (2009) 9:81–98. 10.1080/16184740802461744

[79] Thiel A Meier H. Überleben durch abwehr–zur lernfähigkeit des sportvereins. Sport Gesellschaft. (2004) 1:103–24. 10.1515/sug-2004-0202

[80] UN. Sport for Development and Peace: Towards Achieving the Millennium Development Goals. (2003). Available online at: https://www.sportanddev.org/en/document/manuals-and-tools/sport-development-and-peace-towards-achieving-millennium-development (accessed July 18, 2022).

[81] Engel GL. The need for a new medical model: a challenge for biomedicine. Science. (1977) 196:129–36. 10.1126/science.847460 847460

[82] UNICEF. Flüchtlingscamp Za'atari. (2022). Available online at: https://www.unicef.de/spenden/fluechtlingscamp-zaatari-jordanien (accessed July 18, 2022).

[83] Harrell-Bond B. Can humanitarian work with refugees be humane? Hum Rights Q. (2002) 24:51–85. 10.1353/hrq.2002.001128319151

[84] Doidge M Sandri E. ‘Friends that last a lifetime': the importance of emotions amongst volunteers working with refugees in Calais. Br J Sociol. (2019) 70:463–80. 10.1111/1468-4446.12484 29756402

[85] Feuchter M Janetzko A. Refugees welcome in sports“ - bewegungsangebote für geflüchtete im spannungsfeld zwischen integrationsforderung und partizipationszwang. Sport Gesellschaft. (2018) 15:125–57. 10.1515/sug-2018-0008

[86] Michelini E Burrmann U Nobis T Tuchel J Schlesinger T. Sport offers for refugees in Germany. Promoting and hindering conditions in voluntary sport clubs. Soc Reg. (2018) 2:19–38. 10.14746/sr.2018.2.1.02

[87] Bergholz L Stafford E D'Andrea W. Creating trauma-informed sports programming for traumatized youth: core principles for an adjunctive therapeutic approach. J Infant Child Adol Psychother. (2016) 15:244–53. 10.1080/15289168.2016.1211836

[88] Ley C Lintl E Team MK. Movi kune–gemeinsam bewegen“: bewegungstherapie mit kriegs-und folter-überlebenden. Spectrum. (2014) 26:71–97.

[89] Wacquant LJ. Pugs at work: bodily capital and bodily labour among professional boxers. Body Soc. (1995) 1:65–93. 10.1177/1357034X95001001005

[90] Zander B Thiele J. Jugendliche im Spannungsfeld von Schule und Lebenswelt. Wiesbaden: Springer (2020).

[91] Eikenberry K,. Ten Questions to Ask After Setting Your Goals. (2015). Available online at: https://blog.kevineikenberry.com/leadership-supervisory-skills/ten-questions-to-ask-after-setting-your-goals/ (accessed July 18, 2022).

[92] Giulianotti R Armstrong G. The sport for development and peace sector: a critical sociological analysis. In: Schulenkorf N, Adair D, editors. Global Sport-for-Development. New York, NY: Palgrave Macmillan (2013). p. 15–32.

[93] Adams R. Empowerment, Participation and Social Work 4 Ed. Houndsmills: Palgrave MacMillan (2008).

[94] Kreitzer L. Liberian refugee women: a qualitative study of their participation in planning camp programmes. Int Soc Work. (2002) 45:45–58. 10.1177/0020872802045001319

[95] McGuire-Adams TD Giles AR. Anishinaabekweg dibaajimowinan (stories) of decolonization through running. Soc Sport J. (2018) 35:207–15. 10.1123/ssj.2017-0052

[96] Hammersley M Atkinson P. Ethnography: Principles in Practice 4 Ed. New York, NY: Routledge (2019).

